# Stable solid and aqueous H_2_CO_3_ from CO_2_ and H_2_O at high pressure and high temperature

**DOI:** 10.1038/srep19902

**Published:** 2016-01-27

**Authors:** Hongbo Wang, Janek Zeuschner, Mikhail Eremets, Ivan Troyan, Jonathan Willams

**Affiliations:** 1 Max Planck Institute for Chemistry, Chemistry and Physics at High Pressures Group and Atmospheric Chemistry Department, PO Box 3060, 55020 Mainz, Germany; 2 Institute of Crystallography, Russian Academy of Sciences, Leninsky pr. 59, Moscow 119333, Russia

## Abstract

Carbonic acid (H_2_CO_3_) forms in small amounts when CO_2_ dissolves in H_2_O, yet decomposes rapidly under ambient conditions of temperature and pressure. Despite its fleeting existence, H_2_CO_3_ plays an important role in the global carbon cycle and in biological carbonate-containing systems. The short lifetime in water and presumed low concentration under all terrestrial conditions has stifled study of this fundamental species. Here, we have examined CO_2_/H_2_O mixtures under conditions of high pressure and high temperature to explore the potential for reaction to H_2_CO_3_ inside celestial bodies. We present a novel method to prepare solid H_2_CO_3_ by heating CO_2_/H_2_O mixtures at high pressure with a CO_2_ laser. Furthermore, we found that, contrary to present understanding, neutral H_2_CO_3_ is a significant component in aqueous CO_2_ solutions above 2.4 GPa and 110 °C as identified by IR-absorption and Raman spectroscopy. This is highly significant for speciation of deep C–O–H fluids with potential consequences for fluid-carbonate-bearing rock interactions. As conditions inside subduction zones on Earth appear to be most favorable for production of aqueous H_2_CO_3_, a role in subduction related phenomena is inferred.

Carbon dioxide (CO_2_), when dissolved in water CO_2_(aq), readily reacts to form carbonic acid (H_2_CO_3_), but the acid dissociates so rapidly under ambient aqueous conditions to bicarbonate (HCO_3_^−^) and H_3_O^+^ that its very existence was doubted for a long time^1^. Consequently, undissociated, neutral aqueous H_2_CO_3_(aq) is usually not considered a relevant species in investigations of the CO_2_/H_2_O system in geological applications^2^,^3^. A number of synthetic pathways towards solid H_2_CO_3_ have been explored, including: treatment of cryogenic CO_2_/H_2_O mixtures with high-energy ion irradiation^4^; proton irradiation of pure solid CO_2_ (ref. 5); and direct protonation of HCO_3_^−^ or CO_3_^2−^ in frozen aqueous solutions^6^,^7^,^8^,^9^. Until recently it was believed, that crystalline H_2_CO_3_ could exist as two separate polymorphs, *α*- and *β*-H_2_CO_3_, which could even be resublimed while retaining their polymorphic structure^10^,^11^. However, by employing a novel strategy for the synthesis of solid H_2_CO_3_ through gas-phase pyrolysis of alkyl carbonates, Reisenauer *et al.*^12^ could demonstrate that what was known as *α*-H_2_CO_3_ is actually its monomethyl ester (CH_3_OCO_2_H) rather than the acid itself, leaving the *β*-polymorph as the only observed crystal structure of H_2_CO_3_ to date. Yet, the exact crystal structure of *β*-H_2_CO_3_ is still unknown. The FT-IR and Raman spectra of solid H_2_CO_3_ below 220 K at low pressure are now well established and several theoretical studies have reported on the structure and stability of H_2_CO_3_ with respect to decomposition to CO_2_ and H_2_O as well as on the relative stability of its isomers and on their vibrational characteristics^7^,^8^,^13^,^14^,^15^. Its molecular decomposition rate has been investigated both experimentally^16^ and theoretically and it was found to be accelerated over 10^12^ times at ambient conditions by as few as three water molecules to reach macroscopically observed decomposition rates^1^,^17^. Interestingly, increased thermal stability of solid H_2_CO_3_ at elevated pressure has been predicted by *ab initio* molecular dynamic simulations^18^. However, no indications of metastable states at elevated pressure or increased thermodynamic stability of H_2_CO_3_ (aq) have been found up to 1.94 GPa^19^.

In this work, we studied H_2_O/CO_2_ mixtures experimentally at high pressure and temperatures in a diamond anvil cell (DAC), looking for evidence of increased stability of molecular H_2_CO_3_ in aqueous solution at elevated pressure. Within the Earth, at depths corresponding to pressures of up to 4.6 GPa (~160 km, upper mantle), temperatures are heterogeneously distributed^20^, depending on the exact environment, but can reach 1500 °C when melt is present. To cover this terrestrially relevant temperature range two sets of experiments were performed. In the first set of experiments, H_2_O/CO_2_ mixtures were compressed to the desired pressure and then heated by irradiation with a CO_2_ laser. The laser was focused to irradiate only a fraction of the sample. In this way, the behavior of the system at high *T* can be investigated in the center of the heated area while phenomena of the transition down to room temperature can be investigated on the hot spot periphery. A solid product was synthesized during laser heating and the pressure-dependence of the vibrational modes of this product was investigated up to 9.1 GPa at ambient temperature. Additionally, the stability of the solid product with respect to pressure was investigated by compression to 25 GPa after synthesis at 4.3 GPa. In the second set of experiments, the sample was heated resistively to up to 280 °C after compression to the desired pressure. During resistive heating, the evolution of the system was monitored *in situ* by IR absorption spectroscopy. In both sets of heating experiments, the pressure range from 1.5 to 4.6 GPa was covered.

## Results of laser heating experiments

**Figure 1:**
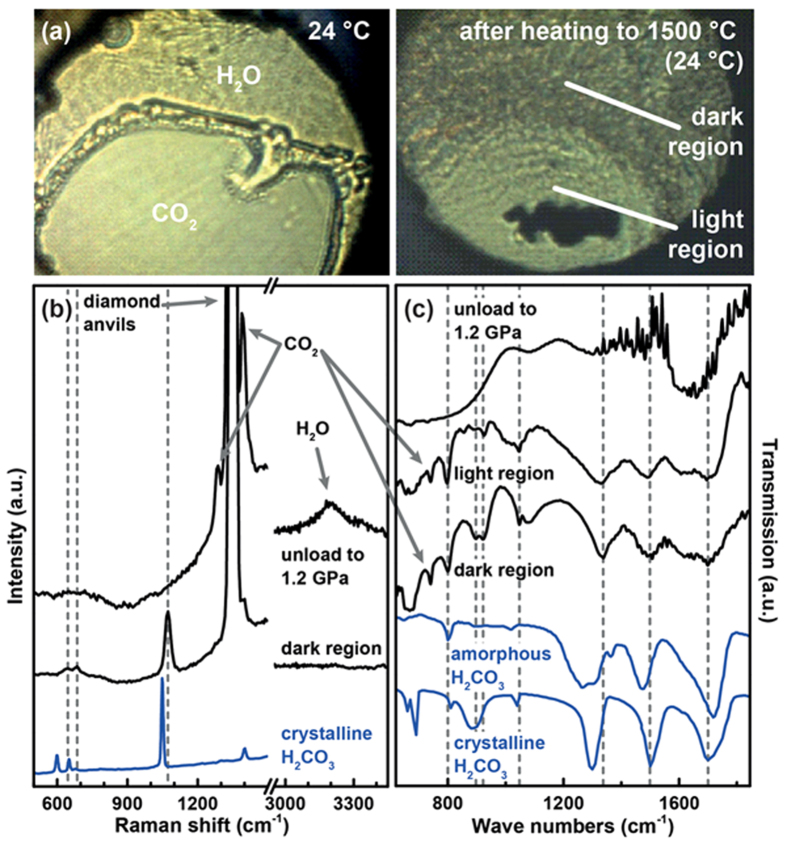
(**a**) Images of the CO_2_/H_2_O mixture before and after laser heating to 1500 °C at 3.5 GPa. The whole sample area was scanned with a CO_2_-laser. The laser was switched off at the light area at the bottom of the image. The black piece in the center of the light area is gasket material (platinum). Raman spectra (**b**) and IR absorption spectra (**c**) of the sample were recorded at 3.5 GPa after cooling down to room temperature from 1500 °C. Reference Raman and IR absorption spectra of crystalline and amorphous H_2_CO_3_ are shown in blue for comparison^8^,^13^. The vertical dashed lines highlight the observed high pressure absorption bands of H_2_CO_3_ while the reference spectra were recorded *in vacuo*. The spectrum recorded at 1.2 GPa shows the decomposition of H_2_CO_3_ after pressure was reduced to below 2.4 GPa where upon pressure dropped by itself to 1.2 GPa.

To simulate conditions inside the Earth, we studied CO_2_/H_2_O mixtures with a wide range of pressures (1.5 GPa, 2.1 GPa, 2.6 GPa, 3 GPa 3.5 GPa and 4.6 GPa) up to 1500 °C by CO_2_ laser heating. The measurement on a CO_2_/H_2_O (1:1.4) mixture at 3.5 GPa shall serve as an example of our results. When the directly illuminated sample area was heated above the melting temperature of the components, only a small part around this area remains liquid due to the steep thermal gradients encountered in laser-heating experiments in a DAC. During laser-heating to about 1500 °C, a dark substance formed around the directly heated area, which disappears again when the laser spot is moved above it. However, the dark substance remained at the trailing end of the scanning movement which enabled us to deposit it in the whole sample area. The directly heated region remained clear after the laser was switched off (Fig. 1a). This transformation is accompanied by a large increase in volume, as indicated by the increase of pressure after laser heating (Supplementary Fig. S1). The ‘dark’ appearance of the product is probably not the consequence of actual light absorption in the visible range but due to a macroscopically heterogeneous sample structure. Quenching of the sample at the trailing end of the moving laser spot likely happens too quickly to allow for proper crystal growth, leaving finely dispersed crystallites of the synthesized product, CO_2_ and H_2_O, possibly within a glassy mixture of all three. Differences in the refractive index of the different components of the mixture will cause internal scattering of incident light and the “dark” appearance in this area. We will continue to use the term ‘dark’ to distinguish the area with synthesized solid product from the rest of the sample.

The Raman spectrum (Fig. 1b) of the dark substance recorded at room temperature showed a sharp, intense peak at 1073 cm^−1^ and two small peaks at 639 cm^−1^, and 685 cm^−1^. Meanwhile, the intensity of the Raman modes of CO_2_–I (1290 cm^−1^, and 1393 cm^−1^) and the O-H stretching mode of H_2_O-Ice VII (3000-3400 cm^−1^) are greatly reduced with the appearance of these new peaks. The only compounds in the CO_2_-H_2_O binary system known to form from a reaction of its endmembers are CO_2_ clathrate hydrates^21^ and members of the carbonate equilibrium. The presence of CO_2_ clathrate hydrate can be excluded as its Raman spectrum^21^ largely resembles the spectra of H_2_O and CO_2_ and even the recently discovered high-pressure modification was shown to decompose above a pressure of 1 GPa^21^. The only member of the carbonate equilibrium from pure H_2_O/CO_2_ mixtures known to exist as a solid is H_2_CO_3_. Indeed, the sharp peak at 1073 cm^−1^ is coincident with the intense mixed mode of the symmetric stretching vibrations ν_s_(C–O) and ν_s_(C=O) of crystalline H_2_CO_3_ (Supplementary Table S1)^8^ while the two weaker peaks at 639 cm^−1^, and 685 cm^−1^ coincide with skeletal vibrations of crystalline H_2_CO_3_, and with its in-plane deformation vibration ν_ip_(COO), respectively^8^. The weak mixed Raman mode of the δ_ip_(COH) and ν(C–O) vibration in crystalline H_2_CO_3_ (ref. 8), usually observed at 1403 cm^−1^, might be obscured by the very strong Raman band from diamond at 1332 cm^−1^ or by the ν_s_(C=O) vibration of CO_2_. Also, the IR absorption spectra of both the dark and the light sample region at room temperature (Fig. 1c) show absorption bands at 800 cm^−1^, 1048 cm^−1^, 1337 cm^−1^, 1498 cm^−1^ and 1699 cm^−1^ in both spectra which are well correlated to the respective bands in reference spectra of amorphous and crystalline H_2_CO_3_ (ref. 13). It is noteworthy that the IR spectrum of the dark region shows a double peak at 896 cm^−1^, and 920 cm^−1^ (highlighted with two dashed lines in Fig. 1c). This feature has been observed for H_2_CO_3_ previously^13^. The presence of intense absorption in this frequency range has been recognized as a marker for the crystallinity of H_2_CO_3_, as can be seen from a comparison of reference spectra from both polymorphs^13^. Experiments on different CO_2_/H_2_O ratios (from excess CO_2_ to excess H_2_O) revealed, that the dark product could be observed at any investigated composition (Supplementary Fig. S2).

**Figure 2:**
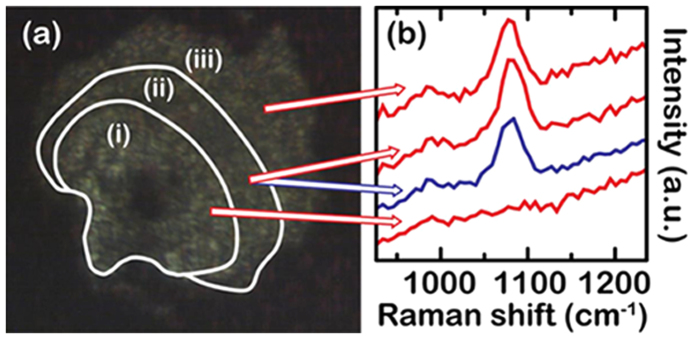
The still image (a) and Raman spectra (b) during laser heating at 4.0 GPa. The arrows indicate the place of measurement – the fluid zones in the inner, directly heated area (i), around the directly heated area (ii), and the solid outer zone (iii). The color of the spectra in (**b**) corresponds to spectra recorded during heating (red) and after heating (blue). The liquid state around the directly heated area has been confirmed by a video recorded during heating (Supplementary Video S1). Region (iii) is solid during the entire experiment.

**Figure 3:**
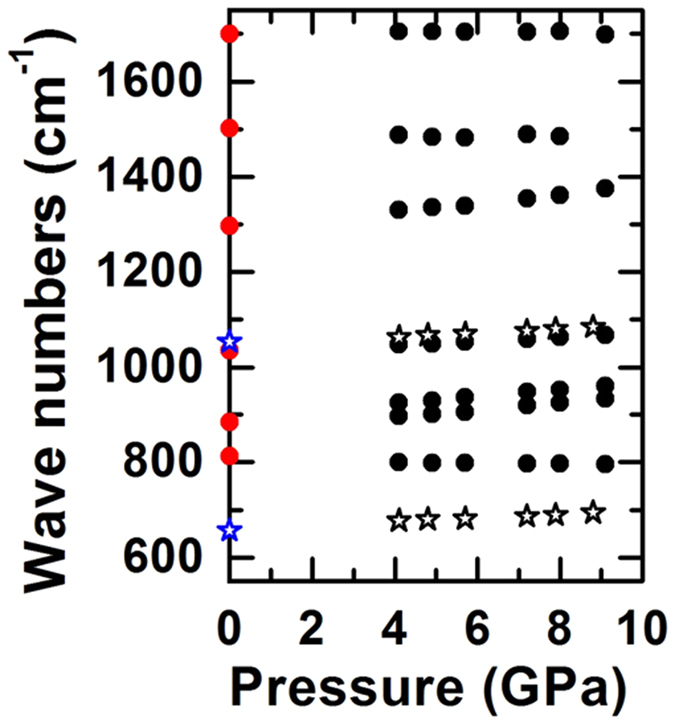
Pressure shifts of Raman modes (black stars) and IR modes (black circles) for a 1:1 CO_2_/H_2_O mixture after preparation of crystalline H_2_CO_3_ by laser heating to 1500 °C obtained by successive compression. Reference data for the positions of Raman modes (blue stars) and IR modes (red circles) for crystalline H_2_CO_3_ are shown at the pressure of the experiment. The weak skeletal Raman mode at 639 cm^−1^ at 3.5 GPa was not detectable during some experiments and the peak position could not be unequivocally be determined in this experiment.

While it is tempting to take the correlations between the observed bands in our experiment and the published spectra of solid H_2_CO_3_ as proof for the presence of H_2_CO_3_ in our experiments, it must be borne in mind that these reference spectra were recorded under cryogenic conditions *in vacuo* (Supplementary Table S1). When comparing the band positions with published data, small differences become apparent: δ_oop_(CO_3_) is found at slightly lower wavenumbers, the skeletal bending mode, δ_ip_(COO), δ_oop_(COH), ν_as_(C–O)/ν_as_(C=O), and δ_ip_(COH) are found at higher wavenumbers than the bands from the reference spectra while ν_as_(C=O), and ν_as_(C(OH)_2_) appear essentially at the same frequency. We are confident that the position of IR absorption bands in H_2_CO_3_ is not very sensitive to temperature as the published IR absorption bands of H_2_CO_3_ from Kohl *et al.*^8^ and Winkel *et al.*^13^ are essentially identical although they were recorded at temperatures differing by 120 K (Supplementary Table S1). To test if the position of the strong 1073 cm^−1^ Raman mode is sensitive to sample temperature we first synthesized the dark product as described above from a CO_2_/H_2_O (1:1.8) mixture at 4.0 GPa and then turned on the laser again at lower power to heat the center of the directly illuminated the sample area to below 700 °C. A video taken during laser heating (Supplementary Video S1) shows that both the heated spot and a circular area around it were liquid while about half the sample away from the heated area remained solid (Fig. 2a). We measured the Raman spectrum of the sample during heating *in situ* in the center of the heated area, at the liquid edge and in the solid exterior (Fig. 2b). The Raman mode at 1073 cm^−1^ could be observed in the liquid and in the solid outer sample area but not in the directly heated inner part. Furthermore, its frequency was invariant, independent of where it was recorded and at which temperature. Hence, the observed differences in Raman band position are likely related to the pressure differences of our experiments to the ones reported in literature. To investigate the pressure dependence of the observed bands, we studied the dependence of Raman shift and IR absorption on pressure, as shown in Fig. 3. In this experiment, we first synthesized the dark product by laser heating a 1:1 CO_2_/H_2_O mixture at about 3.9 GPa, and then successively compressed to 9.1 GPa while recording Raman and IR absorption spectra at 24 °C. With increasing pressure, δ_oop_(CO_3_) displays a red shift, δ_oop_(COH), and ν_as_(C–O)/ν_as_(C=O), and δ_ip_(COH) blue-shift while ν_as_(C=O) and ν_as_(C(OH)_2_) do not shift significantly. Extrapolating to the low pressure at which the reference spectra were recorded, all modes observed in our experiment shift towards the published spectral features of H_2_CO_3_ (ref. 8).

When the whole DAC containing a sample of solid H_2_CO_3_ produced by laser-heating, was heated again resistively to temperatures significantly beyond the melting point of H_2_O at the given pressure, all spectroscopic traces of solid H_2_CO_3_ disappeared and only CO_2_ and H_2_O could be found in the Raman spectrum. The same is true for samples that were depressurized to below 2.4 GPa (Fig. 1b,c). The decomposition upon decompression is accompanied by a pressure drop of similar magnitude as the pressure increase during initial synthesis. The decomposition to H_2_O and CO_2_ is exactly the behavior that is conventionally expected for solid H_2_CO_3_ in contact with liquid water. Considering the spectral features of the product obtained from laser-heating the H_2_O/CO_2_ mixture together with the observed pressure shift of the absorption bands and the reaction behavior upon decompression as well as its dissolution in contact with liquid water, the identification of the solid product from our laser-heating experiments as H_2_CO_3_ is reasonable. Furthermore, the coincidence of the published IR absorption and Raman band positions of H_2_CO_3_ with the extrapolated position of the IR absorption and Raman bands observed in our experiments indicates that the crystal structure of H_2_CO_3_ in our experiments could be identical with the crystal structure of H_2_CO_3_ obtained in cryogenic experiments *in vacuo*, i.e. *β*-H_2_CO_3_ (ref. 8).

Comparable results were found in a number of similar experiments in the pressure range from 2.4–4.6 GPa. Below 2.4 GPa, no evidence for formation of solid H_2_CO_3_ could be found. This indicates a critical role for pressure during formation of solid H_2_CO_3_ during laser heating.

The increasing pressure when solid H_2_CO_3_ is synthesized by laser heating poses the question whether this effect is due to the crystal structure of H_2_CO_3_ having a higher volume-per-molecule than the equimolar amounts of CO_2_ and H_2_O. If this were true, solid H_2_CO_3_ would be expected to be destabilized by increasing pressure. To investigate this, solid H_2_CO_3_ was synthesized from a CO_2_/H_2_O mixture (1:2) initially held at 4.3 GPa as described above. After laser heating, the pressure increased to 5.2 GPa, consistent with earlier observations. The sample was then compressed to 25 GPa while the intense Raman band at 1073 cm^−1^ was recorded (Supplementary Fig. S3). Up to the highest pressure investigated here, no decomposition of the preformed solid H_2_CO_3_ could be observed. Again, consistent with earlier experiments, decomposition was only observed at ambient temperature when pressure was decreased to below 2.4 GPa.

## Results of resistive heating experiments

**Figure 4:**
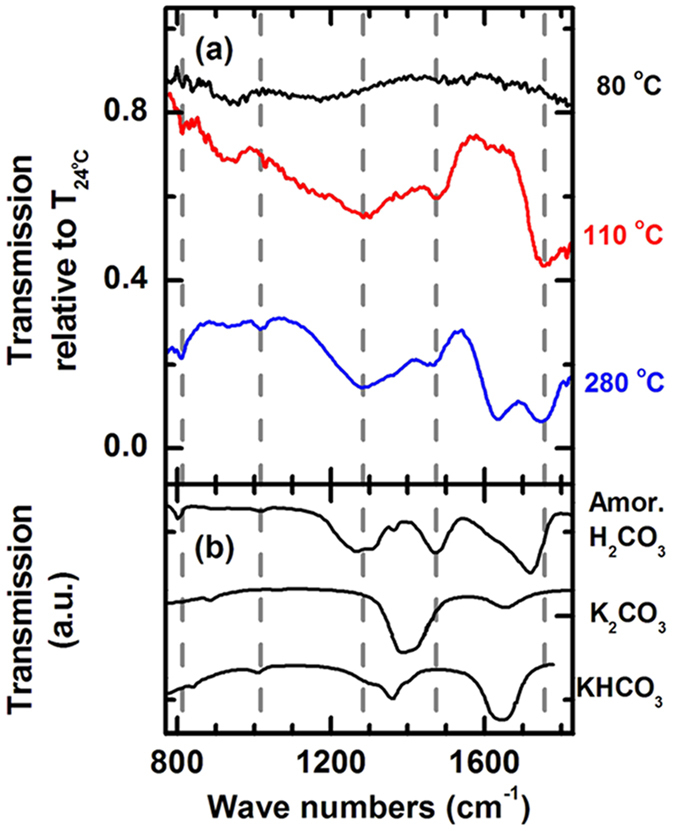
(**a**) *In situ* IR absorption spectra of a 1:1 CO_2_/H_2_O mixture while heating from 24 °C to 280 °C at 2.4 GPa. The displayed spectra have been divided by the initial spectrum recorded at 24 °C (Supplementary Fig. S1). (**b**) Reference spectra of amorphous H_2_CO_3_ (ref. 13) and aqueous solutions of K_2_CO_3_ and KHCO_3_ (ref. 22). The vertical dashed lines show five absorption bands assigned to carbonic acid, all of which are evident in the 280 °C spectrum.

A prerequisite for the precipitation of H_2_CO_3_ during laser heating should be the existence of dissolved molecular H_2_CO_3_ (aq) along the thermal gradient at the edge of the directly heated area. To investigate this, a 1:1 mixture of CO_2_ and H_2_O at 2.4 GPa was resistively heated to 280 °C with a rate of 5 °C/min and the spectral evolution was monitored *in situ* by IR absorption spectroscopy (Fig. 4a). To eliminate the influence of absorption bands from CO_2_ and H_2_O ices, the spectra are referenced to the spectrum of the solid sample at 24 °C by division. The spectrum recorded at 24 °C which was used as reference only shows the combined spectral contributions from CO_2_-I and H_2_O-Ice VI. Upon heating to 80 °C, a weak broad band appeared at about 945 cm^−1^. This minor band is an artifact from the division through the initial spectrum recorded at 24 °C (Supplementary Fig. S4). The spectrum recorded at 110 °C shows the development of a number of new bands at 810 cm^−1^, 1293 cm^−1^, 1483 cm^−1^ and 1746 cm^−1^. These new absorption patterns cannot be explained by pressure-induced shift of bands from CO_2_, H_2_O, HCO_3_^−^ or CO_3_^2−^. Instead, the only member of the global carbonate equilibrium in the CO_2_/H_2_O system that shows such absorption features is H_2_CO_3_, as demonstrated by a reported reference spectrum of amorphous H_2_CO_3_ acquired *in vacuo* (Fig. 1c)^13^. The slight shift of the observed absorption bands as compared to the reference spectrum of amorphous H_2_CO_3_ and to the observed band positions of solid H_2_CO_3_ obtained during laser heating can be explained by the aqueous state of H_2_CO_3_ in this experiment. With the temperature increased to 280 °C, three new absorption bands gradually appear: two weak bands at 1020 cm^−1^ and 1362 cm^−1^, and one strong band at 1634 cm^−1^. The first band can be assigned to H_2_CO_3_ (ref. 13). The main contribution to the bands at 1362 cm^−1^ and 1634 cm^−1^ can be assigned to the ν_s_(CO_2_) and ν_as_(CO_2_) mode of HCO_3_^−^ (ref. 22), respectively, which is known to form when CO_2_ dissolves in liquid H_2_O. The 1634 cm^−1^ band of HCO_3_^−^ is distinctly different from the absorption feature at 1746 cm^−1^ and HCO_3_^−^ has only one absorption feature in this region. This precludes the assignment of the 1746 cm^−1^ band to HCO_3_^−^ (Fig. 4b). An aqueous solution with CO_3_^2−^ present shows only weak absorption in this region^22^ at 1655 cm^−1^ which also cannot explain the absorption band at 1746 cm^−1^ (Fig. 4b). The assumption of the presence of a mixture of H_2_CO_3_ and HCO_3_^−^ in solution and hence a superposition of their spectral features also explains why the band at 1293 cm^−1^ is much broader than it is in the reference spectrum of pure amorphous H_2_CO_3_ (ref. 13). This experiment provides strong evidence that the spectrum at 280 °C in Fig. 4a contains a significant contribution from undissociated H_2_CO_3_, probably in aqueous form. However, in contrast to the laser heating experiments, no precipitation of solid H_2_CO_3_ was observed at any time during heating or cooling down to ambient temperature.

Comparable results were found in a number of similar experiments in the pressure range from 2.4–4.5 GPa. Below 2.4 GPa, even when heating to higher temperatures (up to 250 °C), no evidence for formation of aqueous H_2_CO_3_ could be found. Again, this indicates a critical role for pressure during formation and stability of aqueous H_2_CO_3_.

## Discussion

We have shown that molecular H_2_CO_3_ appears to be an integral component of CO_2_/H_2_O mixtures at and above pressures of 2.4 GPa at elevated temperature. This finding can be rationalized on the following grounds. The relative abundance of H_2_CO_3_ and its dissociation products HCO_3_^−^ and CO_3_^2−^ in an aqueous solution of CO_2_ is largely determined by the dielectric constant (*ε*) and the autodissociation constant *K*_w_ of its host solvent H_2_O, and both are complex functions of pressure (*p*) and temperature (*T*)^23^,^24^. Generally, increasing *p* favors increased ionization, i.e. an increase in *K*_w_ of water. As a result of this, in the carbonate system with increasing *p*, more ionic species (HCO_3_^−^ and CO_3_^2−^) are created from neutral, dissolved CO_2_(aq) due to the larger abundance of H^+^. Isobaric heating of water affects *K*_w_ and thereby the extent of ion pairing of the dissolved members of the carbonate system^23^. As ion pairing of HCO_3_^−^ and CO_3_^2−^ in absence of metal ions can only mean pairing with H^+^, the free acid H_2_CO_3_ will be produced. Indeed, it has been observed that both ionization constants of aqueous H_2_CO_3_ decrease upon heating^25^,^26^. This effect is also observed in other inorganic acids. For example, the pH of a 0.5 mmolal aqueous solution of H_2_SO_4_ was observed to increase from 3.2 to almost neutral pH when heated to 400 °C at 0.4 GPa and similar associative behavior upon heating is observed in aqueous solutions of HCl and H_3_PO_4_ (ref. 27). Within the Earth, increasing pressure and temperature conditions also leads to a lowering of the dielectric constant of water, which makes it a significantly better solvent for electrically neutral species produced from ion pairing. The combination of low *ε* and high *K*_w_ at depth therefore favor the existence of stable electrically neutral H_2_CO_3_ rather than at the surface. The physical reason for the apparent instability/higher reactivity of aqueous H_2_CO_3_ below 2.4 GPa is not yet understood.

It has been shown here that H_2_CO_3_ can precipitate as a crystalline solid from solution during laser heating at the edge of the directly heated area The possibility to produce solid H_2_CO_3_ in a DAC will enable us to investigate its crystal structure in future experiments and it will make the study of the high-pressure behavior of H_2_CO_3_ possible, in first place. One conclusion that can already be drawn from our experiments investigating the pressure dependence of the vibrational modes of solid H_2_CO_3_ observed with Raman and IR absorption spectroscopy is, that the crystal structure of H_2_CO_3_ which is conventionally produced at cryogenic conditions (i.e. *β*-H_2_CO_3_) and H_2_CO_3_ which is produced by the novel high-pressure technique presented herein in the investigated pressure range are likely identical.

The observation of the 1073 cm^−1^ Raman mode in the liquid area around the directly heated part of the sample means that solid H_2_CO_3_ does not dissolve under the high-pressure conditions of our experiment during laser-heating, even in presence of liquid water. This seems to contradict the observation that solid H_2_CO_3_ (produced during laser heating) disappears as soon as liquid water is present (during whole-cell heating). From the latter observation combined with the absence of re-precipitation of solid H_2_CO_3_ during slow cooling after whole-cell heating it can be inferred that solid H_2_CO_3_ is not thermodynamically stable in our experiment in presence of liquid water even at elevated pressure (>2.4 GPa). The formation and metastability of solid H_2_CO_3_ during laser heating must therefore be the result of a non-equilibrium process. Based on the observation of apparent of H_2_CO_3_ molecules at elevated temperatures during whole-cell heating, we suggest the following model for this non-equilibrium process. The concentration of H_2_CO_3_ molecules increases with temperature in CO_2_/H_2_O fluids above 2.4 GPa. By laser heating, a convective motion is initiated in the cell which transports mass away from the heated area, following the steep thermal gradient at its edges. In pure H_2_O, a polar molecule like H_2_CO_3_ would be expected to be miscible at all ratios. However, when CO_2_ is added to aqueous solutions, the ability to dissolve polar/ionic species is decreased due to the lower dielectric constant (ε) relative to pure H_2_O at the same conditions^28^. Many ternary systems of a polar/ionic solute in a CO_2_/H_2_O mixture show an immiscibility gap for high concentrations of CO_2_ and solute with decreasing temperature while the same solute would be entirely soluble in pure H_2_O under the same conditions^29^. Accordingly, when fluid containing high concentrations of H_2_CO_3_ molecules is transported to the cooler edges of the heated area faster than thermodynamic equilibrium can be re-established, H_2_CO_3_ concentrations could be high enough to make solid H_2_CO_3_ a stable phase in the CO_2_/H_2_O/H_2_CO_3_ ternary system. When the laser beam is moved across the sample, solid H_2_CO_3_ that precipitated from the hot fluid at the cooler edges of the heated area is then stabilized by the rapidly solidified CO_2_/H_2_O/H_2_CO_3_ matrix at the trailing end of the heated spot before it can dissolve by reaction with liquid water at lower temperatures. The steep thermal gradients that are apparently needed to produce solid H_2_CO_3_ in a DAC together with the apparent need for stabilization in a solid matrix make it highly unlikely that solid H_2_CO_3_ occurs as an abundant solid inside the Earth. However, the necessity of steep thermal gradients for the synthesis of solid H_2_CO_3_ also emphasizes the large effect rising temperature has on the abundance of dissolved molecular H_2_CO_3_ in deep fluids.

Due to its instability at ambient pressure, H_2_CO_3_ has never been considered significant in models of COH fluids inside the Earth^30^. This simplified approach should be reconsidered in the light of this study. Our experiments indicate that molecular H_2_CO_3_ is a stable species in aqueous fluids above 2.4 GPa (~ 85 km depth), and at least up to 280 °C at 3.5 GPa, and its overall stability field in aqueous fluids is far from being mapped. Some constraints can be put on the formation of H_2_CO_3_ from our experimental results, though. As a classic Brønstedt acid, its stability will depend strongly on pH with high H^+^ concentrations promoting H_2_CO_3_ formation. Its stability will also depend on the redox-state of the environment and is probably limited to regions where CO_2_ is stable, too. Furthermore, our experiments indicate that fluids present at higher temperature will contain more H_2_CO_3_ than cooler fluids.

A limiting factor on the provenance of H_2_CO_3_ will be the simultaneous availability of free CO_2_ and H_2_O. In subduction zones, which probably show the most favorable conditions for formation of H_2_CO_3_ on Earth in terms of pressure, and temperature^31^,^32^, and abundance of free CO_2_ and H_2_O, aqueous carbon bearing fluids are created by devolatilization of the subducting slab^32^. With increasing temperature, the fluids in the subducting slab become increasingly enriched in CO_2_ with the expected peak concentration above 750 °C^32^. Even when assuming a hot geotherm (~225 K/GPa)^32^, the pressure coinciding with the begin of peak CO_2_ concentration in the fluid is well above the minimum pressure of 2.4 GPa identified by us for the stability of aqueous H_2_CO_3_. Production of aqueous H_2_CO_3_ could therefore be connected to subduction related volcanism.

Depending on the amounts of H_2_CO_3_ produced, CO_2_ and H_2_O could be significantly deprived from hot fluids through H_2_CO_3_ formation which has consequences on the geochemistry of these fluids. An apparent “masking” of H_2_O and CO_2_ in fluids at pressure above 2.4 GPa can have an influence on numerous properties of deep fluids like activity of its constituents, electrical conductivity, viscosity and density^33^. On the other hand, decomposition of H_2_CO_3_ below 2.4 GPa would be equivalent to a sudden release of CO_2_ and H_2_O which should be accounted for in models of deep fluids in this pressure region. If H_2_CO_3_ formation happens at the expense of ionic species of the carbonate equilibrium, increased solubility of carbonate minerals can be expected as compared to models neglecting H_2_CO_3_ formation^34^. Future studies on the formation of H_2_CO_3_ molecules in aqueous fluids above 2.4 GPa should concentrate on a quantitative description of the global carbonate equilibrium as all the above mentioned effects will strongly depend on the equilibrium concentration of H_2_CO_3_ under the respective conditions. These results will also be relevant for the geochemical modeling of large, water-rich exoplanets.

## Methods

CO_2_ (purity > 99.99%) was loaded into IIa type diamond anvil cell (DAC) containing ultrapure H_2_O (>18 MΩ) with the aid of a gas loader at 500 bar. The CO_2_/H_2_O ratio was determined from the relative volumes occupied by the then solid CO_2_ and H_2_O, considering the molar volumes at the respective pressure. Typically we used a gasket made of Rhenium or Platinum. The 632.8 nm line of a He-Ne-laser was used to excite the Raman spectra. Infrared (IR) measurements were conducted using a Bruker IFS-66V FTIR spectrometer equipped with a KBr beam splitter and a globar mid-infrared source. The IR beam size was adjusted according to the measured area. Spectra of the dark and the light region were recorded by shading off the part to be excluded from the light source of the spectrometer. High temperatures were achieved either by irradiation with a CO_2_-laser or by means of whole-cell external heating. In external heating experiments, the temperature was determined with the help of a thermocouple glued directly on one of the diamond anvils. In laser-heated experiments, temperature was determined by the glow color in the center of the heated area. While temperature estimation from the color of glowing may produce a big uncertainty, our experiments showed that crystalline H_2_CO_3_ can be synthesized in a wide temperature range as apparent from no visible glowing to a white glowing sample area. This means, that this big uncertainty in temperature has no significant effect on the validity of the results of this study. A ruby chip was inserted in the sample and the pressure determined from the peak wavelength of the *R*_1_ ruby fluorescence band.

## Additional Information

**How to cite this article**: Wang, H. *et al.* Stable solid and aqueous H_2_CO_3_ from CO_2_ and H_2_O at high pressure and high temperature. *Sci. Rep.* **6**, 19902; doi: 10.1038/srep19902 (2016).

## Supplementary Material

Supplementary Information

Supplementary Video
