# Supplementary material for: Stable solid and aqueous H2CO3 from CO2 and H2O at high pressure and high temperature
Source: Sci Rep. 2016 Jan 27;6:19902. doi: 10.1038/srep19902 (PMC4728613; doi:10.1038/srep19902)
Supplement: Supplementary Information [file srep19902-s1.pdf]

Supplementary information for

Stable solid and aqueous H_2CO_3 from CO_2 and H_2O at high pressure and high temperature

Hongbo Wang,[1] Janek Zeuschner,[1] Mikhail Eremets,[1]* Ivan Troyan,[1,2] Jonathan Willams[1]

[1]Max Planck Institute for Chemistry, Chemistry and Physics at High Pressures Group and Atmospheric Chemistry Depertment, PO Box 3060, 55020 Mainz, Germany.

[2]Institute of Crystallography, Russian Academy of Sciences, Leninsky pr. 59, Moscow 119333, Russia.

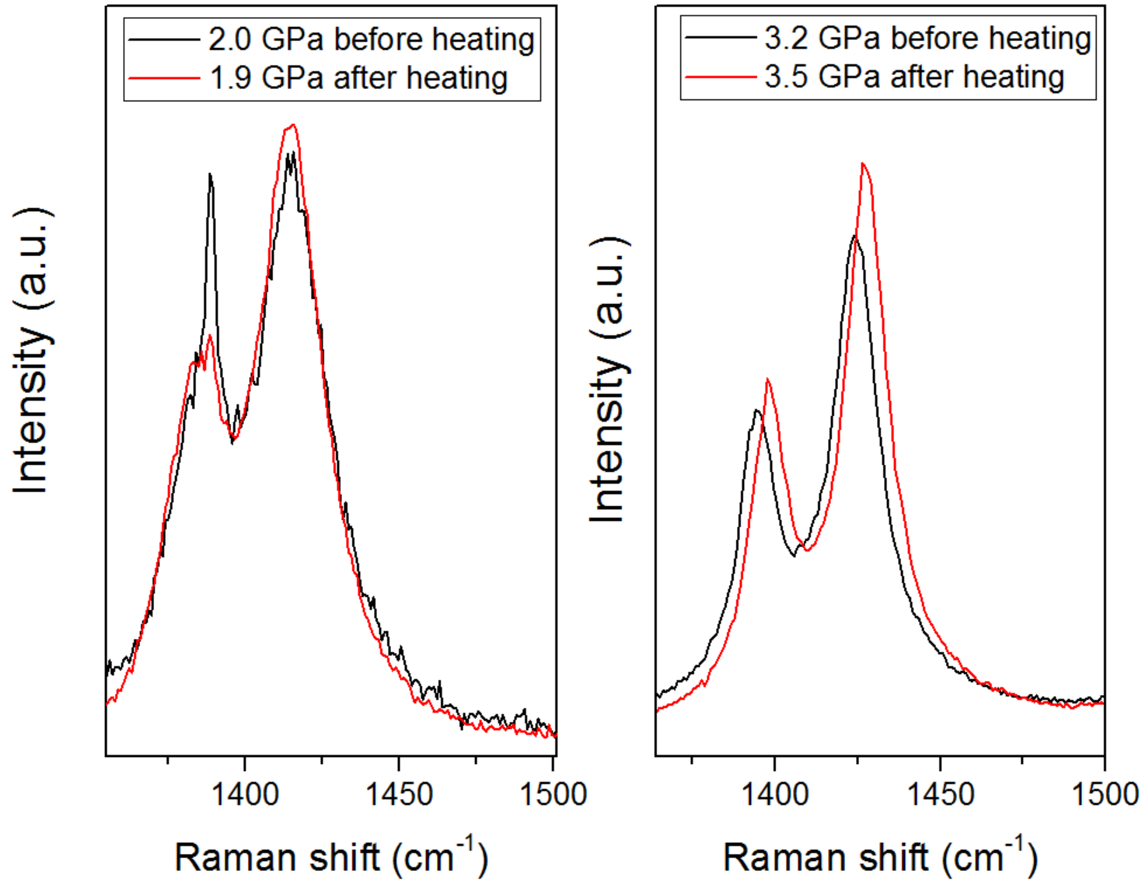

Fig. S1. Raman shift of ruby fluorescence before (black line) and after (red line) laser heating performed on a mixture of CO_2/H_2O (1:3.6) at 2.0 GPa and 3.2 GPa, respectively. After laser heating at 2.0 GPa, the pressure does not show apparent change. However, further compress to 3.2 GPa and heat again, the pressure increase to 3.5 GPa. The auto-increase of pressure indicates a volume increase during the formation of H_2CO_3.

Table S1.Vibrational Raman and IR modes of H_2CO_3 in cm^{-1} and their assignment according to Kohl *et al*. (Ref. 8 in main paper) and Winkel *et al* (Ref. 13 in main paper).

Our work			Kohl *et al*.		Winkel *et al*.	
solid		aqueous	solid		solid	
3.5 GPa		2.4 GPa	0 GPa		0 GPa	Assignment
24 ^oC		280 ℃	-193 ^oC		-73 ^oC	
Raman	IR	IR	Raman	IR	IR	
639			605			Skeletal bend
685			657			δ_{ip}(COO)
	800	810		812	812	$\delta_{oop}(CO_3)$
	896, 920			884	880, 900	δ_{oop}(COH)
	1048	1017		1035	1035	ν_{as}(C-O) and ν_{as}(C=O)
1073			1054			ν_s(C-O) and ν_s(C=O)
	1337	1285		1297	1297	δ_{ip}(COH)
	1498	1464		1502	1501	$\nu_{as}(C(OH)_2)$
	1699	1750		1700	1700	ν_{as}(C=O)

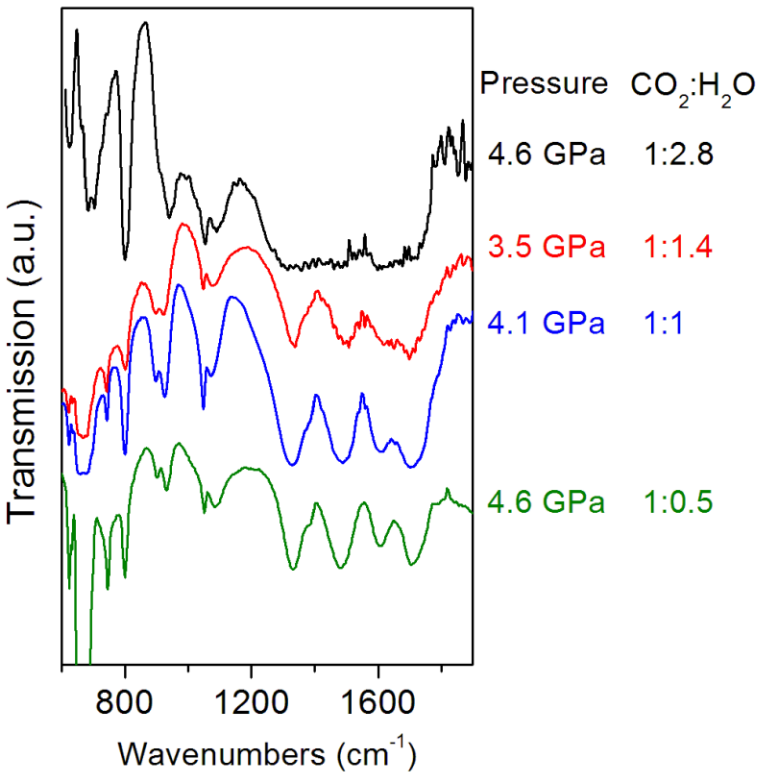

Fig. S2. IR absorption spectra of mixtures with different CO_2/H_2O ratios after heating them to 1500 ℃ with a CO_2 laser. The spectra were recorded at the given pressures after cooling down to room temperature.

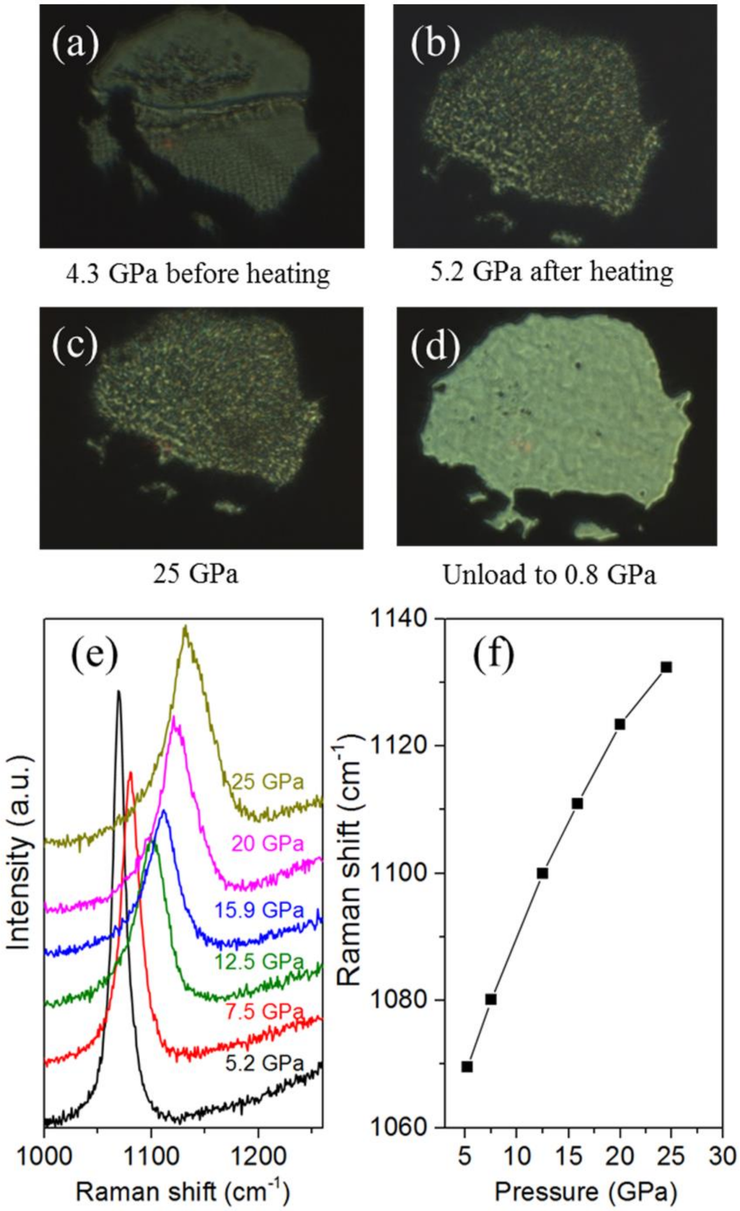

Fig. S3. (a) and (b) show the images of mixture at 4.2 GPa before and after heating, respectively. (c) The image of the sample compressed to 25 GPa after laser heating at 4.2 GPa. (d) The image after the sample was unloaded to 0.8 GPa. (e) Raman spectrum (ν_s(C-O) and ν_s(C=O)) with increasing pressure after the mixture was heated at 4.3 GPa. (f) Pressure dependence of H_2CO_3 Raman vibron (ν_s(C-O) and ν_s(C=O)).

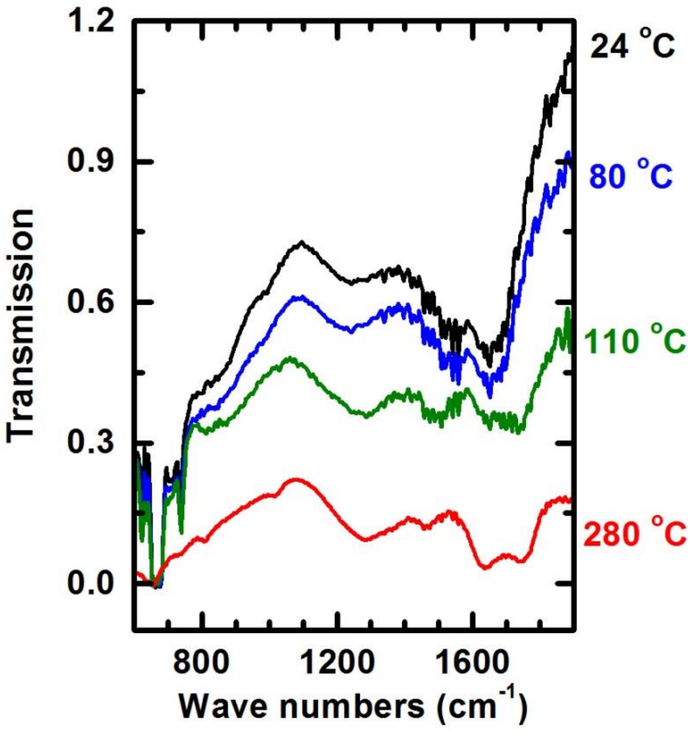

Fig. S4. *In situ* IR absorption spectra used for the spectra shown in Fig. 4a after division through the IR absorption spectrum of the empty DAC.

Caption for Video S1. Video during laser heating at 4.0 GPa. During laser heating, both the heated spot and a circular area around it were liquid while about half the sample away from the heated area remained solid.
